# Navigating Heart–Lung Interactions in Mechanical Ventilation: Pathophysiology, Diagnosis, and Advanced Management Strategies in Acute Respiratory Distress Syndrome and Beyond

**DOI:** 10.3390/jcm13247788

**Published:** 2024-12-20

**Authors:** George E. Zakynthinos, Vasiliki Tsolaki, Kostantinos Mantzarlis, Andrew Xanthopoulos, Evangelos Oikonomou, Konstantinos Kalogeras, Gerasimos Siasos, Manolis Vavuranakis, Demosthenes Makris, Epaminondas Zakynthinos

**Affiliations:** 1 3rd Department of Cardiology, “Sotiria” Chest Diseases Hospital, Medical School, National and Kapodistrian University of Athens, 11527 Athens, Greece; gzakynthinos2@gmail.com (G.E.Z.); boikono@gmail.com (E.O.); kalogerask@yahoo.gr (K.K.); ger_sias@hotmail.com (G.S.); vavouranakis@gmail.com (M.V.); 2 Critical Care Department, University Hospital of Larissa, Faculty of Medicine, University of Thessaly, Mezourlo, 41335 Larissa, Greece; vasotsolaki@yahoo.com (V.T.); mantzk@outlook.com (K.M.); dimomakris@uth.gr (D.M.); 3 Department of Cardiology, University Hospital of Larissa, Faculty of Medicine, University of Thessaly, 41110 Larissa, Greece; andrewvxanth@gmail.com; 4 Cardiovascular Division, Brigham and Women’s Hospital, Harvard Medical School, Boston, MA 02115, USA

**Keywords:** heart–lung interactions, mechanical ventilation, mechanical circulatory support, right ventricle, positive end-expiratory pressure, PEEP, acute respiratory distress syndrome, ARDS

## Abstract

Patients in critical condition who require mechanical ventilation experience intricate interactions between their respiratory and cardiovascular systems. These complex interactions are crucial for clinicians to understand as they can significantly influence therapeutic decisions and patient outcomes. A deep understanding of heart–lung interactions is essential, particularly under the stress of mechanical ventilation, where the right ventricle plays a pivotal role and often becomes a primary concern. Positive pressure ventilation, commonly used in mechanical ventilation, impacts right and left ventricular pre- and afterload as well as ventricular interplay. The right ventricle is especially susceptible to these changes, and its function can be critically affected, leading to complications such as right heart failure. Clinicians must be adept at recognizing and managing these interactions to optimize patient care. This perspective will analyze this matter comprehensively, covering the pathophysiology of these interactions, the monitoring of heart–lung dynamics using the latest methods (including ECHO), and management and treatment strategies for related conditions. In particular, the analysis will delve into the efficacy and limitations of various treatment modalities, including pharmaceutical interventions, nuanced ventilator management strategies, and advanced devices such as extracorporeal membrane oxygenation (ECMO). Each approach will be examined for its impact on optimizing right ventricular function, mitigating complications, and ultimately improving patient outcomes in the context of mechanical ventilation.

## 1. Introduction

The anatomical proximity and location in the same cavity of the heart and lungs mean that changes in lung inflation and intrathoracic pressure significantly impact circulation. This interaction is critical during mechanical ventilation, where positive inspiratory pressures affect blood flow and cardiac function [1]. Ashbaugh et al. introduced the use of positive end-expiratory pressure (PEEP) in 1967 for severe respiratory distress [2]. Since then, the effects of mechanical ventilation on cardiac function, especially the right ventricle (RV), have been extensively studied.

Research conducted in 1981 showed that increasing the PEEP could reduce cardiac output and the mean blood pressure while shifting the interventricular septum leftward, which restricted left ventricular filling [3]. A 1986 study confirmed that PEEP raised pleural pressure and pulmonary vascular resistance, leading to decreased RV volume and stroke volume in various models [4]. These studies indicate that reduced cardiac output during high PEEP levels often results from diminished venous return and altered ventricular contractility [5].

More recent findings have highlighted the adverse hemodynamic effects of PEEP, particularly in patients with circulatory issues like acute respiratory distress syndrome (ARDS). The ART investigators reported that high PEEP strategies were linked to increased mortality and a higher need for vasopressors due to hypotension [6].

The RV is especially sensitive to changes in afterload [7], and mechanical ventilation influences its function by altering intrathoracic and transpulmonary pressures [8,9]. These alterations affect venous return, RV end-diastolic volume, and wall stress, which, in turn, impact RV output [10]. In ARDS, these effects can severely impair RV performance, driven by mechanical ventilation parameters or increased pulmonary vascular resistance from lung pathology [11].

This overview aims to clarify the complex interactions between mechanical ventilation and cardiac function, particularly concerning the right ventricle. It will also address monitoring techniques for assessing the impact of mechanical ventilation on heart function and potential management strategies to mitigate these effects.

## 2. Pathophysiology

The intrathoracic cardiopulmonary vascular system constitutes about 17% of the total vascular volume, with approximately 9% in the pulmonary circulation, which is crucial for left ventricular preload and stroke volume (SV) [11]. Any disruption in pulmonary blood flow directly impacts the SV and overall hemodynamics.

In a healthy state, cardiac chambers and major vessels are influenced by pleural pressure (Ppl), while pulmonary capillaries are affected by transpulmonary pressure (TPP). The pericardium’s rigidity means changes in one ventricle’s volume can impact the other, a concept known as ventricular interdependence [12].

For effective gas exchange, pulmonary blood flow must match ventilation, relying on driving pressure (the mean pulmonary artery pressure minus the mean left atrial pressure) and pulmonary vascular resistance (PVR) [13]. Increased resistance may arise from hypoxic vasoconstriction [14] or hypercapnia [15]. Due to their high compliance, pulmonary vessels can alter diameter and resistance in response to surrounding pressures, affecting blood flow similarly to collapsible tubes. Elevated extravascular pressure reduces the diameter of pulmonary vessels, fully compresses some of them, and results in increased PVR [13].

J.B. West’s model identifies three zones of pulmonary blood flow based on driving pressure and alveolar pressure, which varies during the respiratory cycle [13]. Due to hydrostatic pressure, in zone 3, the blood flow is continuous during the respiratory cycle; in zone 2, blood flow may be compromised if alveolar inspiratory pressure exceeds capillary pressure, while in zone 1, alveolar pressure is higher than capillary pressure, resulting in no blood flow in mechanical ventilation.

Positive end-expiratory pressure (PEEP) and tidal ventilation increase airway pressure (Paw), which is transmitted to Ppl and TPP, thus affecting pulmonary circulation and blood flow [16]. The effects of airway pressure on pulmonary circulation have a direct impact on right ventricular preload. On the other hand, PEEP can also enhance left ventricular function, mostly evident in patients with heart failure, reducing its afterload, provided it does not adversely affect right ventricular performance [11].

The hemodynamic effects of ventilation largely stem from variations in Ppl and TPP. Changes in Ppl primarily influence RV inflow and, as a result, LV outflow, while TPP affects RV outflow and LV inflow [17]. Mechanical ventilation alleviates respiratory muscle strain, allowing cardiac output to meet metabolic demands, but it can induce cardiovascular stress during weaning, potentially leading to heart failure and pulmonary edema [9].

### 2.1. Right Ventricular Dynamics and Preload Influences

Preload refers to wall stress at the end-diastole of the RV. Under normal conditions, the force during RV ejection remains stable, and fluid resuscitation increases both end-systolic and end-diastolic RV volumes. If blood is removed from a hypothetic static circulation, the mean systemic filling pressure (Pmsf) drops, causing the blood volume to fall below the threshold necessary to return blood to the right heart [9].

The blood volume in the systemic circulation below this threshold is termed unstressed volume, while the volume above it is defined as stressed volume. Stressed volume is the active blood volume that creates a gradient for blood return towards the heart. Increasing intravascular volume above the stressed volume raises Pmsf along the venous compliance curve. Different vascular beds possess varying amounts of unstressed volume; redistributing blood flow toward circuits with lower unstressed volumes elevates Pmsf and increases venous return pressure [18,19].

The respiratory cycle directly influences venous return through changes in the pressure gradient by altering the right atrial pressure (Pra) [20]. Pra counters venous return and opposes Pmsf, with fluctuations during the ventilatory cycle leading to reciprocal changes in venous return rates [21]. The transmural pressure for the right atrium is the pressure gradient between Pra and external pressure (Ppl) [9].

During spontaneous (unassisted) breathing, the decrease in Ppl (becoming more negative) results in RA pressure decrease, enhancing venous return and increasing RV end-diastolic volume. Conversely, expiration increases Pra, slightly reducing venous return. Clinical investigations indicate that RV filling occurs below its unstressed volume [9]. Positive-pressure ventilation reverses the typical impact on Pra during the respiratory cycle, leading to elevated Pra during inspiration and decreased levels during expiration.

The pressure gradient responsible for blood flow from venous reservoirs to the heart typically ranges from 4 to 8 mmHg [22], sufficient to maintain cardiac output. Minor increases in PEEP can significantly reduce preload and overall cardiac output.

**Figure 1:**
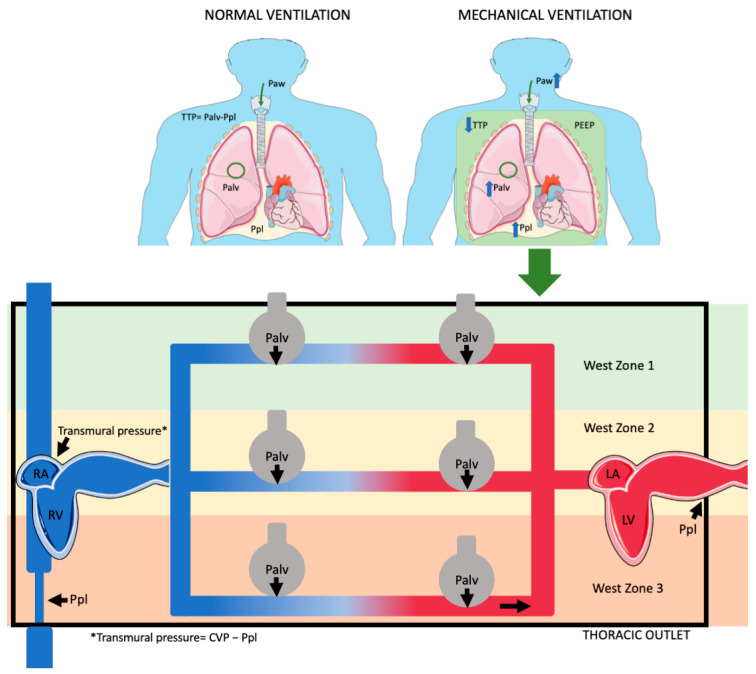
Effects ofositive pressure ventilation on pleural pressure (Ppl), alveolar pressure (Palv), airway pressure (Paw), and transpulmonary pressure (TTP). Ppl, which is generally negative during spontaneous breathing (normal ventilation), becomes less negative or even positive under positive pressure ventilation. Palv, typically negative or zero during normal ventilation, becomes positive during positive pressure ventilation. Similarly, Paw is negative during normal breathing but becomes positive with positive pressure. Palv has a different effect on various West zones. In zone 1, pulmonary vessels fully collapse; in zone 3, they collapse only partially. In zone 2, venous part fully collapses, while arterial part only partially collapses. (Parts of figure were drawn by using pictures from Servier Medical Art. Servier Medical Art by Servier is licensed under Creative Commons Attribution 4.0 Unported License (https://creativecommons.org/licenses/by/4.0/, accessed on 20 October 2024).

During inspiration, both in positive and negative pressure, venous return is enhanced from the abdominal vasculature due to increased abdominal pressure from the caudad diaphragmatic displacement [23]. During mechanical ventilation, this effect helps counteract the decline in RV preload caused by the increase in ITP [9]. PEEP effects on RV preload indicates that the RV operates on the steep portion of the Frank–Starling curve, which is preload-dependent, revealing the presence of concurrent hypovolemia (Figure 1) (Table 1).

### 2.2. Right Ventricular Function, Lung Capacities, and Factors Influencing RV Afterload

The RV is highly sensitive to changes in afterload due to its thin myocardial structure and high compliance. Unlike the left ventricle, which is a pressure generator, the RV primarily functions as a flow generator, ejecting blood at lower pressures into the compliant pulmonary vasculature [13,17]. In mechanical ventilation, ITP fluctuations primarily affect LV afterload. However, lung volume alterations during ventilation significantly modify pulmonary vascular resistance and pressures, affecting right ventricular afterload [17,24]. Given its lower contractile reserve compared to the LV, the RV is more adversely affected by swings in ITP and afterload during the respiratory cycle, particularly in conditions like acute respiratory distress syndrome (ARDS), where hypoxic vasoconstriction and capillary microthrombosis may increase afterload and lead to RV failure [17].

Recruitment maneuvers that reopen collapsed alveoli can decrease pulmonary vascular resistance and facilitate RV ejection. Conversely, the hyperinflation of lung volume increases pulmonary vascular resistance and RV afterload, particularly when using large tidal volumes, converting part of zone 3 to zone 2 or even to zone 1 and increasing dead space ventilation [25,26]. Lung-protective ventilation (tidal volumes in the range of 6–8 mL/kg), usually with lower PEEP levels, mitigate these adverse effects.

In lungs with decreased compliance, achieving the same lung volume requires a higher TTP than in normal lungs. However, the intrapleural pressure (ITP) increases similarly in both normal and low-compliance lungs, as ITP rises linearly with lung volume, even under higher TTP. This leads to a reduction in stroke volume as the lung volume increases due to decreased venous return with increased positive pressure.

In ARDS, elevated pulmonary artery pressure can occur independently of mechanical ventilation, causing increased RV afterload due to hypoxic vasoconstriction and lung injury [27]. In these patients with ARDS, low cardiac output can stem from right ventricular systolic dysfunction, tricuspid regurgitation, or suboptimal preload [7]. Diastolic dysfunction can also lead to elevated pressures in the RV and right atrium, causing organ congestion.

### 2.3. Left Ventricular Function and Factors Influencing Afterload

Afterload is the force opposing blood ejection from the ventricle, influenced by aortic pressure, arterial stiffness, and vascular resistance [28]. Increased aortic stiffness makes the arterial system less accommodating to pulsatile flow. Negative pressure breathing reduces Ppl during inspiration, increasing LV transmural pressure and afterload, while expiration raises pleural pressure, decreasing afterload [9]. Healthy individuals manage these fluctuations without significant impacts on LV function. However, significant ITP reduction from conditions like upper airway obstruction can markedly increase LV afterload, possibly leading to pulmonary edema [29].

Mechanical ventilation, especially with high PEEP or large tidal volumes, raises Ppl and decreases venous return and LV transmural pressure, facilitating LV ejection despite rising arterial pressures. However, increased lung volumes can elevate pulmonary vascular resistance, impairing RV ejection. The interplay of decreased venous return and increased pulmonary vascular resistance may lead to critically low cardiac output, particularly in hypovolemic patients and possibly in congestive heart failure [30]. PEEP application also affects diastolic flow dynamics across the mitral valve [31].

During the weaning process from mechanical ventilation, increased LV afterload and venous return during spontaneous breathing may raise myocardial oxygen demand and increased filling pressure, leading to LV failure and failure in the liberation from ventilatory support [9,17].

### 2.4. The Interconnected Dynamics of the Right and Left Ventricles: A Relationship of Interdependence

The LV and RV function in parallel, interconnected through their shared septum and confined within a rigid pericardial cavity. The end-diastolic volume of the LV is linked to the preload of the RV [32]. This interdependence means that the increased filling of one ventricle can negatively affect the diastolic compliance of the other [9]. For example, in pulmonary embolism, significant RV dilation reduces the LV’s end-diastolic volume. Spontaneous breathing can increase the RV’s stroke volume while decreasing the LV’s stroke volume, but overall cardiac output remains stable.

**Figure 2:**
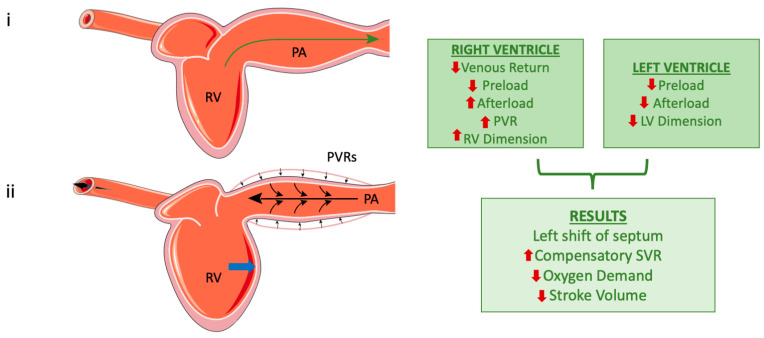
The right ventricle during normal breathing (**top**) and after the application of positive pressure (**bottom**). Subfigure (**i**) represents left ventricle during normal ventilation and the green arrow indicates the direction of blood flow. Subfigure (**ii**). The small black arrows show the compression of the pulmonary artery (PA) caused by positive pressure applied to the respiratory system. The large black arrows represent the backward pressure distribution in the right ventricle (RV) and into the vessels. RV dilation due to elevated pressures [image (**ii**)]; the blue arrow highlights the leftward shift in the midventricular septum, which occurs as a consequence of RV dilation. Finally, the flowchart on the right outlines the effects of positive pressure on both the left and right ventricles, along with their overall systemic impact (the red arrows represent increase or decrease of each parameter). However, these mechanisms are not unique and must always consider the specific conditions of the heart, vascular network, and loading, as well as lung and chest compliance in relation to the mode of mechanical ventilation. (Parts of the figure were drawn by using pictures from Servier Medical Art. Servier Medical Art by Servier is licensed under a Creative Commons Attribution 4.0 Unported License (https://creativecommons.org/licenses/by/4.0/, accessed on 20 October 2024)).

Contrary to previous beliefs that the ventricles operate independently during positive-pressure ventilation, studies have shown that positive-pressure ventilation can influence the LV output due to this interdependence. Reductions in RV dimensions have been associated with increases in LV dimensions, resulting in slight increases in LV stroke volume [33,34]. Contrastingly, RV dilation during MV negatively affects LV cardiac output [35] (Figure 2).

### 2.5. Clinical Applications

The cardiovascular impact of positive-pressure ventilation (PPV) is influenced by mechanical ventilator settings, patient factors, and pharmacological interventions [36,37]. High tidal volumes and low PEEP can lead to significant hemodynamic alterations, such as RV underfilling during inspiration and exaggerated RV output during expiration, resulting in cyclical changes in capillary under- and overfilling, a condition inducing microvascular injury, ultimately resulting in RV dilation and failure [38,39]. Conversely, PEEP application might stabilize RV and LV volumes, preventing vascular shearing and minimizing the risk of RV failure.

Research indicates that abrupt deflation following sustained lung inflation can lead to hypoxemia and microvascular damage, while gradual deflation minimizes adverse effects. Controlled ventilation strategies are essential to safeguard cardiac and pulmonary function [40,41]. Recent studies have highlighted the complex communication among the heart, lungs, and kidneys, emphasizing the role of PEEP in this dynamic [11].

This overview underscores the intricate relationship between right and left ventricular function, mechanical ventilation, and their clinical implications in managing critically ill patients.

**Figure 3:**
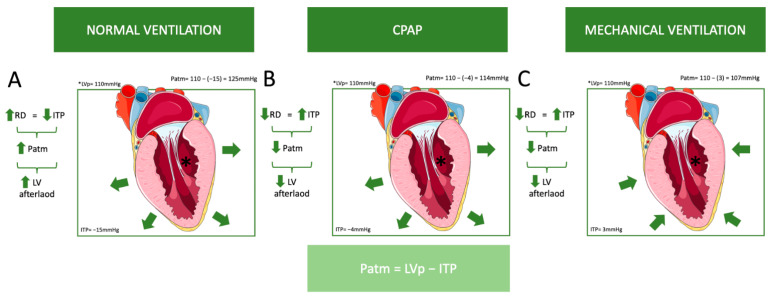
This figure illustrates the influence of different ventilation modes on left ventricular (LV) afterload. As previously mentioned, atmospheric pressure (Patm) is the difference between left ventricular pressure (LVp) and intrathoracic pressure (ITP). (**A**) Normal ventilation: As respiratory drive (RD) increases, intrathoracic pressure (ITP) decreases, which results in an increase in Patm and LV afterload. For example, if LVp = 110 mmHg and ITP = −15 mmHg, then Patm = 110 − (−15) = 125 mmHg. (**B**) Continuous positive airway pressure (CPAP): A decrease in respiratory drive leads to an increase in ITP, resulting in a decrease in Patm and LV afterload. For example, if LVp = 110 mmHg and ITP = −4 mmHg, then Patm = 110 − (−4) = 114 mmHg, which is lower than in normal ventilation. However, ITP remains negative. (**C**) Mechanical ventilation: Similarly, a decrease in respiratory drive leads to an increase in ITP, resulting in decreases in Patm and LV afterload. For example, if LVp = 110 mmHg and ITP = 3 mmHg, then Patm = 110 − 3 = 107 mmHg, which is even lower than with CPAP, and ITP is positive. (The green arrows in the flowcharts indicate an increase or decrease in each parameter. The green arrows around the heart depict the application of pressure on the left ventricle (LV) caused by different ventilation modes.). Abbreviations: CPAP: Continuous Positive Airway Pressure, ITP: Intrathoracic Pressure, LV: Left Ventricle, LVp: Left Ventricle Pressure, Patm: Atmospheric Pressure, RD: Respiratory Drive. (Parts of the figure were drawn by using pictures from Servier Medical Art. Servier Medical Art by Servier is licensed under a Creative Commons Attribution 4.0 Unported License (https://creativecommons.org/licenses/by/4.0/, accessed on 20 October 2024)).

The aforementioned pathophysiology primarily addresses controlled ventilation, but in assisted modes, the dynamics are somewhat different. The presence or absence of respiratory drive, as well as the extent of that drive, can significantly influence hemodynamics depending on the ventilatory mode. In patients with high respiratory drive, assisted ventilation that fails to provide adequate support may mimic the effects of spontaneous breathing rather than delivering the intended benefits of positive-pressure ventilation [36]. This can result in increased negative intrathoracic pressure during inspiration, which may exacerbate venous return, increase right ventricular preload, and elevate left ventricular afterload, potentially worsening hemodynamic instability [36]. Conversely, in assisted modes with appropriately titrated support, the balance between the patient’s respiratory effort and the ventilator’s assistance can mitigate these adverse effects, optimizing hemodynamics by reducing the workload on the respiratory and cardiovascular systems [36]. In controlled modes, where respiratory drive is absent, the hemodynamic effects are primarily determined by ventilator settings, such as tidal volume and PEEP. In the weaning process, where respiratory drive (RD) increases, it has been proven that NIV is very helpful in improving success, as it reduces RD [42]. This distinction underscores the importance of tailoring ventilatory support in assisted modes to the patient’s unique respiratory drive and hemodynamic status (Figure 3).

## 3. Monitoring

### 3.1. Key Indicators and Diagnostic Approaches for Acute Right Heart Syndrome

In mechanically ventilated patients, acute right heart failure (RHF) can present with increasing oxygen demands or sudden cardiovascular collapse [7]. Clinically, RHF may also be accompanied with atrial or ventricular arrhythmias; elevated jugular venous pressure; a gallop rhythm near the left sternal border; or a systolic murmur indicating tricuspid regurgitation, organomegaly, or signs of deep venous thrombosis, particularly in cases related to venous thromboembolism [7]. Persistent weaning failure from mechanical ventilation, often due to the mismatch between right ventricular dysfunction and ventilatory support requirements, is another critical feature of RHF, especially in those with left ventricular systolic dysfunction [43,44].

The role of chest X-rays (CXR) is more aligned with excluding other ICU conditions mimicking RHF, such as pleural effusions, atelectasis, pulmonary edema, or pneumothorax [7].

Electrocardiogram (ECG) findings that can point to right ventricular dysfunction include the presence of a Qr pattern in lead V1, which correlates strongly with myocardial shear stress and elevated troponin levels [45]. Right bundle branch block patients with an R duration exceeding 100 ms in V1 may also demonstrate right ventricular systolic dysfunction [46]. Other ECG signs indicative of right ventricular strain include T wave inversions in leads V1–V4 and the classic S1Q3T3 pattern. Acute Q waves in V1–V3 or right-sided Q waves in V3R–V6R may suggest right ventricular infarction [47]. Despite its specificity, the EKG lacks sufficient sensitivity for an accurate RHF diagnosis [7].

### 3.2. Arterial Catheter—Central Venous Catheter (Hemodynamic Monitoring)

Heart–lung interactions are the mainstay of dynamic fluid responsiveness assessment in mechanically ventilated patients. The change in cardiac output induced by the cycling changes in intrathoracic pressures indicates preload dependence. The most studied tool is pulse pressure variation (PPV). High PPV values suggest that the patient’s stroke volume significantly changes with positive pressure swings, implying fluid responsiveness, but it is less reliable in patients with low lung compliance or tidal volume, which are common in ARDS [48]. Although PPV has limitations in cases of spontaneous breathing, low tidal volumes, and decreased lung compliance, when these conditions are present and PPV remains high (>12–13%), the patient may respond to fluid administration [49]. A tidal volume challenge or a transient increase in PEEP has been shown to enhance PPV’s predictive accuracy by counteracting the drawbacks of low VT ventilation in ARDS [50,51].

Moreover, an increase in the cardiac index of >8.6% during a transient decrease in PEEP (from 12 cmH_2_O to 7 cmH_2_O, referred to as the PEEP-test) in patients without spontaneous breathing predicted volume responsiveness with a sensitivity of 96.8% (95% confidence interval (95%CI): 83.3–99.9%) and a specificity of 84.9% (95%CI 68.1–94.9%). The area under the receiver operating characteristic curve of the PEEP-test for detecting volume responsiveness was 0.94 (95%CI 0.85–0.98) (*p* < 0.0001 vs. 0.5) [52]. End-inspiratory and end-expiratory maneuvers have also been used to assess fluid responsiveness depending on the presence or cessation of heart–lung interactions. End-expiratory occlusion stops cyclic impediment in venous return, and a 4% increase in cardiac output can identify volume responders. If an end-inspiratory occlusion is additionally performed, then the additive change in LV VTI during the two maneuvers can be used to assess fluid responsiveness [53,54].

In severe RV failure, a high PPV could signify RV afterload dependence rather than fluid responsiveness. In such scenarios, clinicians should evaluate RV function through echocardiography or assess PPV changes during passive leg raising (PLR). A decrease in PPV during PLR suggests fluid responsiveness, while no change may indicate RV afterload dependence [17]. Various pulmonary factors, including lung compliance, tidal volume, and pulmonary hypertension, can blunt the predictive power of PPV. In cases of RV failure, further volume expansion can be harmful, as PPV reflects afterload dependency rather than volume responsiveness [55,56,57,58]. Furthermore, the phasic relationship between PPV and stroke volume variation (SVV) during mechanical ventilation is crucial. Importantly, interdependence-induced PPV is generally smaller than the variations seen in preload-responsive patients [59].

Multiple maneuvers have been proposed to evaluate cardiac output, primarily aiming at detecting volume responsiveness. All maneuvers used to assess volume responsiveness require that the patient be fully dependent on mechanical ventilation, in volume control mode, and without arrhythmia.

A central venous catheter (CVC) is often essential in patients with ARDS requiring vasoactive drugs, providing central venous pressure (CVP) measurements. Though CVP is not a reliable predictor of preload responsiveness, trends in CVP values can inform RV function and/or fluid responsiveness. An increasing CVP may indicate declining RV function or volume overload, while a rapid rise in CVP during volume loading without stroke volume improvement points to right heart dysfunction, and further fluid administration should be prevented [60]. Additionally, CVP can offer insights into the RV end-diastolic volume (RVEDV) and pressure (RVEDP), though its accuracy diminishes in conditions such as pulmonary hypertension or myocardial ischemia, where RV compliance is compromised, or when pleural pressure (Ppl) is elevated, which can occur with increased PEEP, especially when lung compliance is high—as in cases like COPD—or when chest wall compliance is reduced, such as in obesity, abdominal compartment syndrome, etc. [44].

A pulmonary artery catheter (PAC) may be useful in diagnostic dilemmas for intricate hemodynamic assessment, despite a decline in its use due to negative clinical findings and the increased use of echocardiography [61]. The pulmonary artery pressure (PAP), pulmonary artery occlusion pressure (PAOP), and pulmonary vascular resistances can be calculated. For patients with severe ARDS and/or shock, advanced hemodynamic monitoring using a PAC was advised in a task force on circulatory shock and hemodynamic management in 2014 [62]. When using high PEEP during ventilation, the PAC helps estimate the true LV filling pressure by calculating the transmural value of PAOP [17]. In ARDS, 24–37% of the PEEP is transmitted to the pleural space [63].

In addition, by using PAC measurements and readings from ventilator waveforms, we can estimate transmural PAOP with relative accuracy.

Transmur PCWP = eePCWP − index of transmission × total PEEP [64].

Index of transmission = eiPCWP − eePCWP/Plateu pressure − total PEEP [64].

(ee = end-expiratory and ei = endinsiratory).

It also enables the monitoring of cardiac output and mixed venous oxygen saturation (SvO_2_), critical for evaluating therapeutic responses.

Increased RV afterload, common in pulmonary disorders, can worsen heart–lung interactions [56,58]. The PAC’s ability to monitor RV dysfunction is valuable, especially with the ratio of CVP to pulmonary artery occlusion pressure [56]. A higher CVP than PAOP signifies RV failure and may lead to adverse cardiac output effects [65]. The continuous evaluation of mixed venous oxygenation aids in assessing hypoxic pulmonary vasoconstriction, often indicated by elevated TTP gradients [55,56]. While the PAC’s thermodilution technique limits immediate stroke volume change identification, newer models with shorter response times may help address this issue [66].

RHF is suspected with RA pressures > 8–10 mmHg or when the RA to pulmonary capillary wedge pressure ratio is ≥0.8, especially with a low cardiac index. If the RV–pulmonary artery gradient exceeds 25 mmHg, echocardiography should assess RV outflow tract obstruction [67]. Right heart catheterization in PAH and suspected RHF cases aids in evaluating left-sided heart disease and the roles of PVR and SVR in treatment decisions and monitoring the response to treatment [68]. In patients with existing PAH, reduced pulmonary artery pressure may indicate worsening RV function [47].

### 3.3. Evaluation of Heart Function in Critical Illness Using Echocardiography

Pulmonary artery catheters (PACs) and echocardiography (ECHO) are vital for diagnosing RV failure and assessing treatment responses in the ICU [69]. ECHO has particularly influenced ventilator management decisions, notably during the COVID-19 pandemic, when ARDS and heart–lung interactions garnered significant attention [70]. While transthoracic echocardiography (TTE) is non-invasive and reliable, transesophageal echocardiography (TEE) can provide better visualization when TTE is limited due to poor echogenicity, especially for detecting acute cor pulmonale [71].

An early echocardiographic assessment is crucial to quickly gather data on ventricular dimensions and function. Key goals include the initial assessment and serial monitoring of the RV size and the function and presence of increased PVRs, including the monitoring of the inferior vena cava size and flow in critically ill patients. Understanding whether RV dysfunction stems from pressure overload, impaired contractility, or a combination of both is essential [71].

Assessing the RV size during ARDS is critical; the RV end-diastolic area (RVEDA) can be compared to the left ventricular end-diastolic area (LVEDA) for evaluation. An RVEDA/LVEDA ratio greater than 0.6 indicates moderate RV dilatation, while a ratio exceeding 1 signifies severe dilatation [72]. Acute cor pulmonale (ACP) is characterized by the presence of a dilated RV (ratio > 0.6) and paradoxical septal motion during end-systole. The echocardiogram should also evaluate the LV ejection fraction, LV end-diastolic area, cardiac output, and markers of LV filling pressures. The limitations of TTE in mechanically ventilated patients include imaging difficulties due to high levels of PEEP, low accuracy in patients with pre-existing cardiopulmonary conditions, and operator dependency [73].

Innovative ECHO techniques enhance the monitoring of heart–lung interactions. Our research group has applied advanced echocardiographic methods to assess RV and LV function in mechanically ventilated patients with COVID-19 ARDS [74]. We utilized Simpson’s method (2D) and 3D echocardiography for LV systolic function evaluation. While both approaches yielded valuable insights, 3D echocardiography provided a more comprehensive volumetric assessment. For RV assessment, we employed 2D speckle tracking echocardiography (2D-STE) to measure RV longitudinal strain (RV-LS)—one of the most sensitive indicators of RV dysfunction. We also calculated RV fractional area change (RVFAC), tricuspid annular plane systolic excursion (TAPSE), and the systolic velocity of the tricuspid annulus (RV S′), along with right ventriculoarterial coupling using the TAPSE/PASP ratio [74].

Among these parameters, RV-LS and the TAPSE/PASP ratio emerged as the most reliable indicators for monitoring RV failure [74]. RV-LS demonstrated significant impairment in most of our cohort, allowing for the early detection of dysfunction even with a normal RV ejection fraction (RVEF). The TAPSE/PASP ratio was essential for assessing RV–pulmonary artery (PA) coupling, correlating closely with patient outcomes. Although LV strain was less sensitive than RV-LS, it helped identify subtle myocardial dysfunction masked by normal EF values, underscoring the importance of multiple assessment methods for comprehensive cardiac function evaluation in mechanically ventilated patients. More importantly, interventricular interdependence was revealed with changes in PEEP [74].

Our findings align with other studies demonstrating that RV-LS measurement via STE is feasible, reproducible, and sensitive for detecting RV dysfunction. Abnormal RV-LS (>−20%) correlates with increased 30-day mortality in patients with COVID-19 on mechanical ventilation [75]. Beakley et al. showed that while the mean RV systolic function defined by the RV FAC (28.9 ± 10.6%) and RV VTI (14.3 ± 4.2%) was diminished, TAPSE (20 ± 4.8 mm), RV S′ (13.5 ± 3.8 cm/s), and RV free wall strain (RVFWS) (−24.1 ± 6.9%) remained preserved [76]. They identified a higher proportion of RV dysfunction via the RV FAC and RV VTI compared to TAPSE, RV S′, and RVFWS [76]. Additionally, the RV FAC was significantly correlated with biomarkers like BNP and highly sensitive troponin I (hs-TnI), indicating that RV-PA coupling provides critical insights beyond standard RV performance metrics in this cohort.

Recent studies highlight that ECHO in mechanically ventilated patients should assess both the anatomy and function of the right heart [69]. The apical four-chamber (A4C) view is crucial for evaluating right heart size and geometry. However, measurements depend on a clear visualization of the RV free wall and lateral tricuspid annulus. While RVEF calculations from 2D ECHO are no longer recommended for clinical use, 2D ECHO remains valuable for assessing RV function, enabling straightforward measurements of the RVFAC and TAPSE, which yield similar information to RVEF [77]. TAPSE and RVFAC do not require geometric assumptions and can be obtained even with suboptimal image quality [78]. Furthermore, RVOT velocity–time integral (RVOTVTI) has proven useful for detecting RV dysfunction in patients with COVID-19, with reduced RVOTVTI (<19 cm) observed in approximately 85% of critically ill patients, while RV free wall strain was reduced in only 30% [76].

Assessing RV-PA coupling using RVFAC/RV systolic pressure (RVSP) and TAPSE/RVSP indices can enhance the monitoring of RV function in patients with COVID-19 [79]. A standard cutoff of 1.0 for normal function identified 86% of hospitalized patients as having RV-PA uncoupling, with significant uncoupling (RVFAC/RVSP < 0.6) detected in 50% of ICU patients [76]. RV myocardial contractile ability also reflects the relationship between RV pressure load and changes in geometry and size. Integrative approaches combining parameters that account for RV afterload are especially useful for evaluating RV function [80]. The RV load–adaptation index (LAIRV) offers a straightforward method to assess this relationship via ECHO, integrating the RV load and cavity dilation, and considering right atrial (RA) pressure [81]. The LAIRV can be computed using TR velocity–time integral (VTITR) and the RV end-diastolic area (AED), making it an easily calculable index from a single A4C view. A small RV area relative to the long-axis length in patients with high RV systolic pressure and low RA pressure indicates a high LAIRV, suggesting good adaptation and RV contractility. Conversely, a large AED relative to the long-axis length indicates poor adaptation [78]. In summary, interpreting RV function is complex, and utilizing various modalities can be beneficial. Further research is necessary to establish a standardized RV evaluation protocol combining measures such as RV-LS, RV FAC, and RV-PA coupling.

An evaluation of LV function in sepsis patients is also crucial to characterize the presence of septic cardiomyopathy. Our study group also demonstrated alternative ECHO methods for recognizing septic cardiomyopathy in shock patients, emphasizing echocardiography’s significance. In our septic shock study, we explored afterload-adjusted echocardiographic parameters to identify and classify septic cardiomyopathy (SC) in mechanically ventilated patients. We found that LVEF and left ventricular outflow tract velocity–time integral (VTI) inversely correlated with SVR, providing insights into SC severity [82]. Afterload-adjusted LVEF and VTI proved valuable for understanding SC phenotypes and monitoring fluctuations in cardiac output during septic shock [82].

Echocardiography is valuable in patients under mechanical circulatory support. In severe cases of RV afterload mismatch, RV output can decrease significantly despite normal intrinsic RV contractility, as seen in acute cor pulmonale. In such situations, treating the underlying cause of impaired gas exchange using V-V ECMO alone may be inadequate since recirculation is exacerbated by reduced RV ejection and TR [83]. ECHO can guide ECMO candidate selection, optimize support strategies, and inform weaning decisions post-cardiopulmonary recovery [69]. Myocardial strain imaging through STE provides sensitive and reproducible measures of myocardial function, offering valuable insights into cardiac performance in patients under mechanical ventilation and mechanical circulatory support [66].

The importance of point-of-care (PoC) ECHO in managing patients with undifferentiated shock in emergency settings has also been highlighted. Additionally, new methods for estimating fluid status have emerged. The Venous Excess Ultrasound Score (VEXUS) is a novel approach that assesses a patient’s volume state based on the IVC diameter, hepatic, portal, and renal vein flow patterns [84,85]. While promising, this tool requires further validation in critically ill patients.

In non-ventilated patients, in order to evaluate fluid load and fluid dependance, the inferior vena cava (IVC) is evaluated. On the contrary, in ventilated patients, these markers for fluid responsiveness can be applied with limitations. Veillard-Baron A et al. demonstrated that a threshold of superior vena cava (SVC) collapsibility of 36% allowed for discrimination between responders and non-responders, with a sensitivity of 90% and a specificity of 100% [86]. Collapsibility of 12% of the inferior vena cava allowed for the identification of responders with positive and negative predictive values of 93% and 92%, respectively [87] (Table 2).

### 3.4. Monitoring Esophageal Pressure for Cardiac Function

Esophageal pressure (Pes) serves as a valuable surrogate for Ppl, as the esophagus is located within the thoracic cavity and reflects pleural pressure changes. Using a small balloon catheter, Pes measurement provides a reliable approximation of Ppl [88]. Monitoring Pes offers insights into the mechanical properties of the lungs and chest wall, which is especially beneficial for evaluating cardiac function. Changes in Ppl affect the pressures around the heart and great vessels, influencing both right and left ventricular performance, as already mentioned (Figure 1 and Figure 2) [88].

Additionally, Pes aids in monitoring TPP changes, which increase pulmonary vascular resistance and right ventricular afterload [88]. Concerning the LV, Pes is crucial for assessing the balance between pleural pressure and LV outflow. Elevated Ppl imposes increased pression on the LV, reducing transmural pressure and oxygen consumption for a given arterial pressure, which may enhance LV efficiency and cardiac output, particularly in afterload-dependent scenarios like left ventricular failure. Thus, monitoring Pes is useful in guiding ventilation strategies and hemodynamic management in complex critical care settings such as ARDS [88]. When using high PEEP during ventilation, the PAC allows for an accurate estimation of the true left ventricular filling pressure by calculating the transmural value of PAOP (transmural pressure = PAOP − esophageal pressure). This method is more accurate than the previously mentioned equations; however, the equations provide a faster approach.

Bi-Ventricular Pressure–Volume Loop Recording, while invasive, is a promising method for assessing right and left ventricular function and their interdependence. It allows for an observation of how changes in one ventricle impact the other, particularly under conditions like pulmonary hypertension or heart failure. A recent study by Dam Lyhne et al. on pigs revealed that right ventricular parameters are more frequently affected than those of the left ventricle, highlighting the need for standardized respiratory conditions in future pressure–volume measurements [89] (Table 3).

## 4. Management

Once RV failure ensues, it triggers a harmful cycle of systemic hypotension, RV ischemia, and dilation, leading to rapid hemodynamic decline. To improve cardiac output from a compromised RV, clinicians can increase the heart rate, decrease RV afterload, enhance RV contractility, or decompress the RV through volume removal [90]. Our research emphasizes identifying RV limitation [35,74]; clinicians should halt fluid loading and consider alternative treatments like inhaled pulmonary vasodilators to boost RV output [88].

### 4.1. Fluid Therapy

While studies indicate that the RV responds better to preload increases than the left ventricle [91], fluid management in ARDS is a great challenge. The RV ejection fraction relies on preload, and hypovolemia can impair the output. Optimizing preload through the Frank–Starling mechanism may improve RV function [68].

On the other hand, fluid loading can worsen pulmonary edema and lead to cor pulmonale [92].

Assessing fluid responsiveness in patients with ARDS is not easy, as low tidal volumes and respiratory system compliance compromise the magnitude of heart–lung interactions and thus the magnitude of changes in indices evaluating fluid responsiveness.

Recent studies advocate for cautious volume resuscitation, emphasizing volume loading only in patients with clearly decreased fluid status, as indicated by hypoxemia, elevated lactate levels, and an increased need for vasopressors [93].

The validity of CVP as a guide for fluid therapy continues to be debated, as already mentioned. A systematic review encompassing 24 studies illustrated a weak correlation between CVP and intravascular fluid status, highlighting the inadequacy of CVP or delta-CVP in predicting the hemodynamic response to fluid challenges [94,95]. Depending on a patient’s position on the Frank–Starling curve, some individuals may be adequately resuscitated at a CVP of 6–7 mm Hg, while others may remain intravascularly volume depleted at a CVP of 10 mm Hg [67]. A recent meta-analysis by Marik et al. revealed insufficient data to support the common practice of utilizing CVP to evaluate intravascular fluid status and guide therapy [96]. In cases of RHF, further volume loading can lead to RV overdistension, increased ventricular interdependence, diminished left ventricular (LV) filling, RV ischemia, and worsening shock [93]. In our opinion, it should be noted that studies do not consider the transmural pressure of CVP but rather the absolute measured value. If transmural CVP was taken into account, the results would likely differ and provide a better assessment of intravascular fluid status.

To maintain a consistent RV preload, the transmural CVP (CVP-Pes) must increase, particularly in situations of rising pleural pressure. While this can be achieved through fluid loading, clinicians should remain alert to avoid abrupt and sustained CVP elevations, as increased CVP can harm other organs, including the kidneys and liver [97]. A hallmark of RV limitation is the increase in CVP from fluid loading without a corresponding rise in stroke volume, which is invariably detrimental [90].

To streamline fluid management, the ARDS Network developed the ‘FACTT-lite’ protocol for patients with ARDS who are not in shock [98]. This protocol has shown similar ventilator-free days compared to the original FACTT protocol. Once an adequate intravascular volume is established, clinicians should monitor for ACP, which is seen in 20–25% of patients with ARDS [98,99]. Supporting the RV is crucial in these cases.

Ultimately, accurately assessing fluid responsiveness is vital for enhancing RV function through fluid administration, combining clinical, hemodynamic, and echocardiographic findings to inform treatment decisions effectively.

When evaluating dynamic indexes of fluid responsiveness in ventilated patients, it is essential to consider several factors that can influence their reliability. The mode of ventilation plays a critical role, as controlled mechanical ventilation with consistent tidal volumes enhances the reliability of dynamic indices such as stroke volume variation (SVV) and pulse pressure variation (PPV) [36]. These indices are reliable only in fully sedated patients on VC mode with a tidal volume of approximately 10 mL/kg, in sinus rhythm, and not in right ventricular dysfunction. This is because, due to the displacement of the interventricular septum, the tests may yield falsely positive results. However, in assisted or spontaneous breathing modes, the variability in intrathoracic pressure changes can reduce the accuracy of these indices [36]. Additionally, the amount of tidal volume is a key determinant, as low tidal volumes, commonly used in lung-protective ventilation strategies for ARDS, may not generate sufficient heart–lung interactions to produce meaningful changes in these indices [9]. The ratio between heart rate and respiratory rate is another important consideration; when the heart rate significantly exceeds the respiratory rate, the overlap of cardiac and ventilatory cycles can distort the expected hemodynamic variations, further compromising the reliability of these measurements [59]. Therefore, clinicians must interpret dynamic indexes of fluid responsiveness in the context of these parameters, adjusting their clinical judgment accordingly to avoid inappropriate fluid management decisions.

### 4.2. Pharmaceutical Treatment

In the context of RV failure, systemic hypotension plays a critical role in reducing perfusion to the right coronary artery, necessitating treatment with vasopressors. The ideal vasopressor should enhance systemic arterial pressure while maintaining or minimizing effects on pulmonary arterial pressure [91]. However, at elevated doses, vasopressors and inotropes can lead to adverse effects, such as tachycardia and myocardial ischemia, and should be reserved for cases of inadequate oxygen delivery after addressing RV preload, afterload, and ischemia.

#### 4.2.1. Vasopressors

1. Norepinephrine and Epinephrine:

Norepinephrine is effective in significantly improving RV function by elevating the mean arterial pressure and increasing RV blood supply, particularly under conditions of high RV wall stress [17]. In patients with sepsis with pulmonary arterial hypertension (PAH) and RV dysfunction, norepinephrine enhances systemic pressure through alpha-1 receptor activation and potentially improves the RV oxygen supply/demand ratio. However, this benefit may be countered by a rise in PVRs and RV afterload at higher doses (>0.5 mcg/kg/min) [46]. Additionally, norepinephrine can improve RV–pulmonary artery coupling and cardiac output through beta-1 receptor stimulation [100].

In cases where RV contractility is severely compromised, such as following RV infarction or cardiopulmonary bypass, epinephrine may be an alternative option due to its stronger alpha-adrenergic stimulation [91].

Phenylephrine, a selective alpha-1 agonist, is not recommended in this context due to the risk of increased PVR [101].

2. Vasopressin:

Arginine vasopressin has a role in managing RV dysfunction and may reduce PVR. Low-dose vasopressin (0.033–0.067 U/min) promotes pulmonary arterial vasodilation by stimulating nitric oxide release, making it beneficial in cases of vasodilatory shock, particularly in patients unresponsive to norepinephrine [46,102]. In postoperative scenarios, where patients might not respond well to catecholamines, vasopressin combined with inotropic agents serves as an effective initial treatment for those with depressed RV function and systemic hypotension [91]. However, at high doses, it can lead to pulmonary and coronary artery vasoconstriction [103].

#### 4.2.2. Inotropes

1. Dobutamine:

Dobutamine, a beta-1 receptor agonist, is often the first-line inotropic agent for RV contractile dysfunction in acute respiratory distress syndrome (ARDS). Its addition can enhance hemodynamics more effectively than volume loading alone or other treatments like nitroprusside or norepinephrine [91]. At low doses (2–5 mcg/kg/min), dobutamine increases cardiac index and stroke volume while decreasing PVR and SVR [46,47,104]. However, higher doses (>10 mcg/kg/min) can induce tachycardia, elevate oxygen consumption, and increase PVRs, necessitating the use of vasopressors [47,104]. In patients with septic shock unresponsive to fluid loading, dobutamine or epinephrine can improve RV contractility despite an increase in mean pulmonary artery pressure [7].

2. Phosphodiesterase (PDE) III Inhibitors:

PDE III inhibitors, such as enoximone and milrinone, enhance myocardial contractility while causing systemic and pulmonary vasodilation by elevating cyclic adenosine monophosphate (cAMP) levels, thereby reducing pulmonary artery pressures and improving RV function in patients with ARDS due to pressure overload [46]. Though they may cause systemic hypotension, they are particularly effective in improving cardiac output and lowering pulmonary pressures in patients with RV dysfunction post-cardiac surgery [91]. Milrinone is often the inotrope of choice in cases of RV failure linked to high pulmonary afterload.

3. Levosimendan:

In our investigation of heart–lung interactions, we found that levosimendan—a calcium sensitizer—holds promise in enhancing hemodynamics in septic cardiomyopathy (SCM). Our study revealed that patients treated with levosimendan experienced a significant increase in LVEF and a notable reduction in lactate levels within the first 24 h despite their severe condition. This suggests that while therapies in septic shock often emphasize left ventricular function, treatments like levosimendan may also positively impact RV dysfunction, contributing to improved outcomes during critical illness [105].

Levosimendan operates independently of the beta-adrenergic pathway, improving cardiac contractility without raising myocardial oxygen demand. Its vasodilatory effects reduce RV afterload, making it particularly advantageous for patients with concurrent pulmonary hypertension [106]. Additionally, inhaled levosimendan has demonstrated benefits in mitigating mortality and inflammatory responses in ventilator-induced lung injury models [107]. Thus, although levosimendan has been evaluated in septic shock often focusing on the effects on left ventricular function, future studies might also evaluate its effects on RV function.

#### 4.2.3. Vasodilators

The right ventricle is highly sensitive to increases in afterload, which can impair performance even with minor increases in pulmonary pressures [91]. Selective pulmonary vasodilation offers a strategy to improve RV function by alleviating afterload without inducing systemic hypotension. Two categories of agents exist for this purpose: intravenous medications with selective pulmonary effects and inhaled agents.

Inhaled nitric oxide (5–10 ppm) and inhaled prostacyclin (20–30 ng/kg/min) are commonly used vasodilators that may improve oxygenation, although their effects on clinical outcomes remain inadequately tested [108]. Inhaled nitric oxide is recommended for short-term therapy to enhance PaO_2_/FiO_2_ ratios in ventilated patients with RHF secondary to ARDS and may stabilize patients with RHF due to massive pulmonary embolism until definitive treatments can be applied [7,109].

Sildenafil, a PDE-5 inhibitor, augments cGMP signaling and can improve symptoms and hemodynamics in patients with chronic pulmonary arterial hypertension, although its acute effects require further investigation. Small studies suggest that PDE-5 inhibitors may mitigate rebound pulmonary hypertension after inhaled vasodilator therapy [110].

Nitric oxide is often preferable to sildenafil in acute settings for several reasons. Firstly, its inhaled route of administration ensures targeted pulmonary vasodilation without systemic hypotension, a crucial consideration in critically ill patients with RHF where maintaining systemic perfusion is essential [109]. Secondly, nitric oxide has an immediate onset of action, making it ideal for rapid stabilization in acute scenarios such as ARDS or massive pulmonary embolism. In contrast, sildenafil, administered orally or intravenously, requires time to take effect and may be associated with systemic side effects, including hypotension [109]. Moreover, nitric oxide’s effects can be precisely titrated and terminated quickly, offering greater control in dynamic clinical situations [110]. However, pulmonary vasodilators can also blunt hypoxic pulmonary vasoconstriction, thereby worsening ventilation–perfusion matching. Pulmonary vasodilators are currently not approved for use in critically ill patients with RV failure not due to PAH [111]. Nevertheless, inhaled nitric oxide (iNO), used off-label, with its rapid onset of action, short half-life, and inhaled route of administration, is the pulmonary vasodilator of choice for critically ill patients and has been shown to improve pulmonary hemodynamics in RHF. Inhaled prostacyclin analogs have also been shown to be safe and effective pulmonary vasodilators for cardiothoracic surgical patients with pulmonary hypertension, refractory hypoxemia, or right heart dysfunction, and they may offer substantial cost savings over iNO [111]. Thus, while both agents play important roles in managing pulmonary hypertension, the unique pharmacokinetics and pulmonary selectivity of nitric oxide make it a superior choice for acute right heart failure in critically ill patients [111].

#### 4.2.4. Diuretics

In RV volume overload scenarios, diuretics can contribute to acute kidney injury due to low renal perfusion rather than renal congestion (leading to decreased glomerular filtration), known as cardio-renal syndrome. Continuous veno-venous hemofiltration (CVVH) may offer greater clinical benefits compared to aggressive diuretic therapy in patients resistant to diuretics [112].

Diuretic use in hypovolemic hypertensive patients undergoing mechanical ventilation may exacerbate heart–lung interactions. Increased PEEP can lead to RV dysfunction and decreased venous return, potentially worsening patient outcomes. The detrimental effects of diuretics in hypertensive patients on mechanical ventilation, particularly with increased PEEP, remain a hypothesis requiring further investigation due to the lack of substantial evidence in this area [113].

### 4.3. Rhythm Control

In addition to achieving optimal filling of the RV, ensuring synchronization between the right atrium and the RV is crucial. For patients experiencing right ventricular infarcts who require the placement of a temporary electronic pacemaker, establishing atrioventricular synchrony can significantly enhance cardiac output compared to disorganized rhythms, and in some cases, it may even reverse states of hypotension and shock. In individuals with right ventricular dysfunction accompanied with a right bundle branch block, positioning the pacing promotes synchronized right ventricular contraction, which may further amplify the advantages of ventricular pacing [114].

However, these techniques have not been evaluated in patients with arrhythmias and RV failure resulting from ARDS or increased pressures due to mechanical ventilation.

### 4.4. Optimizing Ventilation Strategies for ARDS: Balancing Lung and Heart Protection

PEEP is a very important tool in the treatment of ARDS and is often a life-saver in critically ill patients. By preventing alveolar collapse and improving oxygenation, PEEP plays a central role in maintaining adequate gas exchange and reducing the risk of ventilator-induced lung injury. Its application is crucial for stabilizing patients with severe respiratory distress, offering a chance to support lung recovery while optimizing oxygen delivery to vital organs [115]. Additionally, it is generally important to ensure that the tidal volume is set to correct values, which are recommended in the range of 4 to 6 (up to 8) mL/kg body weight, to further minimize the risk of lung injury and maintain optimal ventilation.

In addition to the tidal volume, the driving pressure is an important parameter to monitor during mechanical ventilation. The driving pressure is the difference between the plateau pressure and PEEP, and it has been identified as a strong predictor of ventilator-induced lung injury and mortality. Lower driving pressures are generally recommended, with a target of less than 15 cmH_2_O, as this is associated with better outcomes and reduced lung injury. It is essential to tailor the PEEP and tidal volume settings to minimize driving pressure, optimizing lung protection during mechanical ventilation [46,116].

Hypoxia and hypercapnia cause pulmonary vasoconstriction, increasing RV afterload. Therefore, mechanical ventilation should aim to maintain normoxia and normocapnia with lung- and heart-protective strategies, although hypercapnia cannot be avoided in severe ARDS with very low lung compliance [46,116].

PVR changes may be influenced by lung volume variations. Low volumes, particularly low PEEP, can cause vessel collapse if atelectasis occurs and lung volume falls below the FRC, while high volumes stretch alveolar vessels [7]. Pressure-controlled ventilation can yield similar or better cardiac outputs compared to volume-controlled ventilation with lower tidal volumes [9,117]. In acute lung injury patients, both modes are comparable if the mean airway pressure (Paw) is similar [115], while lung hyperinflation affects cardiac output [118].

Effective hemodynamic management should alleviate RV overload, and mechanical ventilation settings should try to avoid lung derecruitment [119], overdistension [120], and vasoconstriction from hypoxia and hypercapnia [17,121]. Four risk factors for RV failure in ARDS include pneumonia, a PaO_2_/FiO_2_ ratio < 150 mmHg, a driving pressure ≥ 18 cmH_2_O, and PaCO_2_ ≥ 48 mmHg [99]. The presence of all four correlates with over a 60% risk of RV failure.

PEEP optimization enhances alveolar patency while reducing RV afterload. Adequate PEEP prevents derecruitment, but excessive PEEP can cause overdistension and impair RV function [122,123]. High PEEP levels (>15 cmH_2_O) can worsen RV systolic function [124]. Schmitt et al. found that high PEEP levels (13 ± 4 cmH_2_O) increased RV afterload and worsened dysfunction [116].

In contrast, PEEP can reduce right ventricular afterload and improve oxygenation when it successfully resolves atelectasis, thereby recruiting pulmonary vessels. Therefore, a very thoughtful approach should be taken when setting the PEEP level, considering hemodynamic, echocardiographic, and respiratory factors (PO_2_, FiO_2_, lung and chest compliance, etc.).

Individualized treatment is essential, as ARDS can manifest variably among patients. The Surviving Sepsis Campaign-COVID-19 guidelines and the American Thoracic Society (ATS) have recommended treating COVID-19 ARDS according to ARDSnet protocols (high PEEP and low tidal volume). However, our research group has observed that COVID-19 ARDS may not conform to these typical patterns. In patients from our unit, the median static compliance was measured at 52 mL/cm H_2_O, aligning with anecdotal reports of compliance rates between 50 and 65 mL/cmH_2_O across Greece and other countries [125]. Our cohort exhibited a mean PaO_2_/FiO_2_ value of 89; had we adhered to the suggested protocols, a PEEP of 18 cmH_2_O would have been indicated. In contrast, a median PEEP of 8 cmH_2_O was deemed optimal based on a combination of respiratory variables (compliance, functional residual capacity, and PaCO_2_) and hemodynamics as assessed through echocardiography (RV function and systolic pulmonary artery pressure through tricuspid regurgitation) [125]. Increased PEEP levels led to worsened hemodynamics and elevated vasopressor requirements [125].

These findings stem from our research on COVID-19-induced acute respiratory distress syndrome (CARDS), which poses a unique challenge due to its association with a higher prevalence of RV impairment compared to ARDS from other causes [35]. Recent data suggest that improved respiratory mechanics and hemodynamics can be achieved with lower PEEP levels than those recommended by existing guidelines. A prospective study on mechanically ventilated patients with CARDS found that a significant reduction in PEEP (approximately 27.6%) resulted in enhanced RV dimensions and function, characterized by a reduced RV end-diastolic volume, an increased RV ejection fraction, and improved longitudinal strain. Notably, the RV afterload decreased significantly, underlining the importance of respiratory system compliance in predicting cardiac outcomes [35]. The study also indicated that patients in the early intubation subgroup showed more pronounced RV dysfunction, with significant improvements in RV size and hemodynamics following PEEP de-escalation, highlighting the interplay between mechanical ventilation settings and RV performance in CARDS [35].

Our findings have also demonstrated that a PEEP de-escalation resulted in improved respiratory compliance and reduced hypercapnia without negatively impacting oxygenation [126]. This suggests a reduction in lung overdistension, as PEEP reduction led to decreased dead space ventilation and hypercapnia without signs of lung derecruitment (PaO_2_/FiO_2_ remained stable) [126]. Additionally, hemodynamic improvements and better fluid management were also noted [126].

Moreover, COVID-19 ARDS patients exhibit an increased incidence of pulmonary embolism (PE) due to pulmonary vascular endothelialitis and thrombosis, contributing to elevated PVR and RV dysfunction, particularly in ICU settings despite anticoagulation therapy [127]. Mechanical ventilation with high PEEP can exacerbate RV dysfunction by increasing PVR, and lowering PEEP levels may enhance hemodynamics and reduce RV dilation [127].

Vigorous spontaneous breathing should be avoided as forceful inspiratory efforts can increase transvascular pressure and risk lung edema [17]. The ARDSnet trial showed a decrease in ARDS-related hypoxemia from 61% to 25%, highlighting the effectiveness of lung-protective strategies—such as tidal volumes of 6–8 mL/kg predicted body weight (PBW), low plateau pressures, and appropriate PEEP—in reducing ARDS incidence [128].

In patients with ARDS, permissive hypercapnia must be approached cautiously, as acute hypercapnia can worsen pulmonary vasoconstriction and RV dysfunction [129]. However, in some cases of ARDS with low lung compliance, it is necessary to prevent an excessive increase in the driving pressure of mechanical ventilation. In addition, increasing the PEEP can lead to acute hypercapnia, impairing RV function and cardiac index, so lung-protective ventilation should always be accompanied with RV function monitoring [121].

For postoperative cardiac patients, passive ventilation strategies optimize pulmonary function and hemodynamics, especially when combined with ECMO and intra-aortic balloon pumps [130]. High-frequency oscillatory ventilation (HFOV) may recruit collapsed lung units and improve RV function as rescue therapy [10]. Yet the effects of HFOV on RV function have been considered one of the main reasons for treatment failure [131]. Asynchronous ventilation and pendelluft may impair pulmonary circulation and RV function. Prone positioning (PP) improves ventilation uniformity and may unload the RV. Two studies showed that PP restored RV function in overloaded patients [132,133], although our data indicate no changes in cardiac output or pulmonary pressures when comparing supine and prone positions [134]. An improvement in oxygenation after PP was linked to a reduced shunt fraction rather than hemodynamic changes, suggesting that PP is safe for patients with RV failure [134].

Integrating various ventilatory techniques, like prone positioning and ECMO, is vital for managing critically ill patients with ARDS. Individualized treatment is essential. For instance, spontaneous breathing trials without inspiratory support or PEEP can improve LVOT gradients in patients with dynamic LVOT obstruction [37]. In preload-dependent conditions (e.g., right ventricular failure), titrating PEEP can reduce RV afterload. Conversely, positive pressure ventilation benefits patients with reduced ejection fraction and aortic insufficiency by decreasing LV afterload.

Guidelines for mechanical ventilation during VV-ECMO from the Extracorporeal Life Support Organization provide frameworks for clinical settings. The EOLIA and CESAR trials suggest lung-protective settings, including a plateau pressure ≤ 30 cmH_2_O, FIO_2_ ≤ 0.60, and PEEP ≥ 10 cmH_2_O. While the tidal volume is not specified, these guidelines imply a limit of ≤6 mL/kg of ideal body weight for lung protection. If a patient shows higher lung compliance, the ventilator should be adjusted to target a tidal volume of approximately 6 mL/kg to minimize driving pressure and ensure heart-protective ventilation [135].

However, the role of PEEP is also important for the function of the LV. Based on the above, the PEEP may initially appear more like an adversary to the hemodynamic stability of critically ill patients rather than a therapeutic ally. However, its beneficial effects on transmural LV pressure, a critical physiological mechanism which has been described previously (Figure 2), deserve further emphasis, as they underpin its utility in specific clinical scenarios, such as cardiogenic pulmonary edema. By increasing intrathoracic pressure, the PEEP reduces the LV transmural pressure (the difference between intracavitary pressure and the surrounding thoracic pressure), effectively lowering afterload and decreasing the work required by the failing left ventricle to eject blood [29,30]. This reduction in afterload can improve cardiac output while simultaneously alleviating pulmonary congestion by decreasing pulmonary venous return and capillary pressures [30].

These effects are particularly valuable in cardiogenic pulmonary edema, where CPAP (a non-invasive ventilation mode applying PEEP) is routinely recommended and shown to improve oxygenation and reduce dyspnea [29]. Additionally, the ability of PEEP to support ventricular function and prevent further pulmonary fluid accumulation illustrates its dual respiratory and cardiovascular benefits. CPAP also helps open atelectasis in patients with edema, which further aids in improving lung compliance and oxygenation. Mechanical ventilation, similar to CPAP, also contributes to this effect but must be performed with caution [29]. Both CPAP and mechanical ventilation require careful titration and monitoring, as excessive pressure or volume can lead to detrimental effects. For instance, if the PEEP increases too much, it may result in increased dead space in the lungs, which reduces the efficiency of ventilation and gas exchange [30]. This is particularly concerning if the patient has been made hypovolemic through diuretics as part of pulmonary edema treatment. In such cases, the increased dead space can worsen the imbalance between ventilation and perfusion, further compromising oxygenation. These interventions can be monitored by observing the increase in lung compliance, which helps assess their effectiveness [29]. When carefully titrated, PEEP is not merely a respiratory intervention but a multifaceted tool that can optimize hemodynamics in specific clinical contexts, offering a balanced approach to both oxygenation and cardiac performance. Including this perspective highlights the nuanced role of PEEP, which, while potentially detrimental in certain cases, remains an indispensable part of managing conditions like cardiogenic pulmonary edema.

PEEP settings should not follow a one-size-fits-all approach but rather be tailored to each individual patient [136]. Current evidence strongly advocates for personalized PEEP adjustments based on comprehensive respiratory monitoring. Factors such as lung compliance, oxygenation, driving pressure, and the risk of overdistension or derecruitment must be carefully considered when determining the optimal PEEP level [136]. Personalizing PEEP allows clinicians to strike a balance between improving oxygenation and minimizing the risk of ventilator-induced lung injury. This patient-specific strategy ensures that PEEP settings are aligned with the unique physiological needs and responses of each patient, particularly in conditions like ARDS, where lung heterogeneity further complicates the choice of appropriate PEEP levels [136].

### 4.5. Mechanical Circulatory Support (MCS)

In critically ill patients, MCS may become necessary when conventional therapies fail to stabilize hemodynamics. Among MCS options, veno-arterial extracorporeal membrane oxygenation (VA-ECMO), with or without additional support from the LV-Impella or RV-Impella, has emerged as a potential intervention in select populations. The 2019 European Society of Cardiology (ESC) guidelines for acute PE recommend VA-ECMO (Class IIb) in combination with catheter-directed thrombolysis (CDT) or surgical embolectomy for patients with refractory circulatory collapse or cardiac arrest [101]. Additionally, the 2022 ESC guidelines on pulmonary hypertension recommend MCS, particularly VA-ECMO (Class IIa), due to its wider use as an option for selected patients as a bridge to transplantation or recovery [137]. The clinical use of intra-aortic balloon counterpulsation (IABP) in cardiogenic shock remains controversial despite its physiological rationale as it enhances coronary perfusion in both the left and right ventricles. Yet, evidence supporting its efficacy in RV or biventricular failure is insufficient, often necessitating escalation to more advanced MCS [101,138,139].

The complexity of RV failure lies in its multifaceted pathophysiology, often driven by increased PVR or elevated downstream pressures. The decision to implement an RV assist device (RVAD) hinges on the failure to manage RV afterload or filling limitations. It is critical to avoid RVAD in cases where elevated left ventricular end-diastolic pressure (LVEDP) plays a dominant role as this may exacerbate pulmonary edema. Instead, addressing the elevated LVEDP, often with an LVAD, is more effective in these scenarios [90]. Additionally, careful volume management is required, as removing excess volume could also reduce the pressure on the RV if the elevated LVEDP stems from RV dysfunction.

#### 4.5.1. Extracorporeal Membrane Oxygenation (ECMO)

Extracorporeal membrane oxygenation (ECMO) presents a powerful tool in managing complex cardiopulmonary interactions, particularly in ARDS with right heart failure. Venovenous ECMO (VV-ECMO) has been increasingly used in cases of life-threatening hypoxemia, providing respiratory support while also unloading the RV by limiting pulmonary artery hypertension [140,141]. However, the choice between VV-ECMO and VA-ECMO depends heavily on the patient’s underlying condition. VA-ECMO, unlike VV-ECMO, can provide both respiratory and circulatory support, particularly in patients with cardiogenic shock due to severe left ventricular dysfunction [142]. Studies demonstrated that VV-ECMO can significantly reduce pulmonary artery pressures and improve RV function in patients with ARDS. In a study of 13 patients with severe respiratory failure, pulmonary artery pressures dropped immediately after the initiation of VV-ECMO, even before ventilator settings were altered, followed by a decrease in CVP and an increase in CI under stable vasopressor dosing. This emphasizes the crucial role that both oxygenation and decarboxylation play in RV unloading [65,141].

Despite these benefits, VV-ECMO may not always suffice in patients with combined respiratory and circulatory failure. In cases where VV-ECMO fails to alleviate RV dysfunction, transitioning to a veno-arterial-venous (VAV) ECMO mode by adding an arterial cannula might be necessary. Studies have indicated that most patients with ARDS can be initiated on VV-ECMO, and only a small percentage of patients in shock (4.1%) required conversion to VA-ECMO [65]. Additionally, a separate study reported no conversions to VA-ECMO in patients with ARDS on vasopressors after starting VV-ECMO [65]. Nevertheless, if RV failure progresses, despite VV-ECMO, this likely indicates that mechanical support for the left heart or other therapeutic interventions are needed. We recommend starting VV-ECMO for respiratory failure, even in the presence of RV failure, with VAV-ECMO or other circulatory support being reserved for refractory cases [65].

The interaction between ECMO support and the cardiopulmonary system is complex, as changes in lung mechanics and hemodynamics under ECMO differ significantly from conventional management strategies. VV-ECMO allows for ultra-protective ventilation with tidal volumes as low as 2.5 mL/kg, significantly lowering PVR and right heart afterload [143]. Furthermore, careful hemodynamic monitoring is vital, particularly in patients on VA-ECMO, where fluid balance and extracorporeal blood flow can drastically affect outcomes. A positive fluid balance has been associated with poor outcomes, whereas hypovolemia may lead to cannula complications such as hemolysis and reduced flow [144,145]. Optimizing fluid management with small boluses and vasopressors, while monitoring parameters like mean arterial pressure (MAP) and central venous oxygen saturation, helps mitigate these risks [144].

In cases of isolated RV failure, VA-ECMO is becoming less common, as newer percutaneous devices like RV-Impella or percutaneous RVAD offer alternatives. However, VA-ECMO remains a valuable option for biventricular failure as it supports both the RV and systemic circulation without being impacted by RV pressures [146]. Its capacity to unload the RV while maintaining systemic flow and oxygenation is crucial in patients with severe RV afterload issues due to conditions such as pulmonary embolism or ARDS [101,141].

Various research underscores the importance of the personalized titration of ECMO blood flow in preventing right heart failure in patients with ARDS. It is demonstrated that as ECMO blood flow increases, pulmonary artery compliance improves, and the RV stroke work index decreases, indicating reduced workload on the right heart [147]. This highlights how tailored ECMO settings can enhance both pulmonary and cardiac outcomes in these critically ill patients.

The Delphi process conducted by an international expert collaboration has further advanced the management of RV injury (RVI) in patients on ECMO. This process, led by the PRORVnet steering committee, brought together cardiologists, intensivists, and ECMO specialists to develop a consensus on RVI management, emphasizing multimodal diagnostics and unified treatment strategies [148]. The use of echocardiography, including parameters like the RV end-diastolic area (RVEDA) and RV free wall longitudinal strain, is recommended for assessing RV function in these patients.

Left atrial veno-arterial extracorporeal membrane oxygenation (LAVA-ECMO) is an emerging mechanical circulatory support (MCS) strategy, particularly for patients with cardiogenic shock (CS) [149]. LAVA-ECMO involves the use of a multifenestrated transeptal catheter to provide the simultaneous venting of both the left and right atria, offering robust cardiocirculatory support [149]. This approach includes the insertion of a pigtail vent from the right atrium to the left atrium, a form of transmural atrial venting, which helps reduce left ventricular afterload and improves hemodynamics. A recent single-center retrospective analysis of 68 patients (median age of 63 years) who underwent LAVA-ECMO between 2018 and 2023 demonstrated substantial improvements in hemodynamics within 24 h after cannulation, including reductions in right atrial pressure, pulmonary artery pressure, pulmonary capillary wedge pressure, and left ventricular end-diastolic pressure. Indications for LAVA-ECMO included myocardial infarction, biventricular failure, and valvular heart disease [149]. The procedure is performed under intracardiac or transesophageal echocardiographic guidance, with cannulation sometimes involving transcaval access. Although LAVA-ECMO appears to be a safe procedure with no complications from transeptal cannulation, the post-cannulation complications remain high. Survival to decannulation was 69.1%, and the 30-day survival rate was 51.5% [149]. However, further studies are needed to fully assess the safety profile of LAVA-ECMO compared to other MCS strategies, especially regarding its efficacy in providing simultaneous left and right atrial drainage and improving invasive hemodynamics in patients with severe cardiogenic shock [149].

#### 4.5.2. Right Ventricular Assist Devices (RVADs)

Right ventricular assist devices (RVADs) can be surgically implanted or delivered percutaneously, impacting flow in rotary-flow mechanical circulatory support (MCS) devices, which rely on preload and afterload. Flow (Q) is directly proportional to the impeller’s rotations per minute (RPMs) and inversely related to the pressure gradient between the inlet (preload) and outlet (afterload), known as the pressure head (H). In left ventricular assist devices (LVADs), the H is the lowest during systole and highest during diastole, resulting in peak flow during systole [150].

RVADs operate similarly but exhibit less pronounced flow variations due to smaller pressure differences between the right atrium (RA) and pulmonary artery (PA) [150]. For example, they improve heart–lung interaction. In right heart failure, MCS systems cannulate the RA and PA. For patients with acute ischemia, a minimal pressure differential can lead to high flow rates, while severe pulmonary hypertension may reduce flow at fixed RPMs.

Percutaneously delivered assistive mechanical circulatory support (AMCS) devices, such as venoarterial extracorporeal membrane oxygenation (VA-ECMO), TandemHeart, and Impella RP, allow for early intervention without surgery. Both the Impella RP and TandemHeart RVAD bypass the RV, while VA-ECMO displaces blood from the RA to the femoral artery [150].

In isolated RV failure, activating the Impella RP or TandemHeart RVAD reduces RA pressure and increases the mean PA pressure, enhancing the LV preload. In contrast, VA-ECMO may decrease both RA and PA pressures, lowering the LV preload and increasing the LV afterload, potentially reducing native cardiac output [150].

For biventricular failure, the Impella RP and TandemHeart RVAD similarly reduce RA pressure and increase the LV preload. Without LV support, cardiac output may remain unchanged, risking pulmonary edema. VA-ECMO can initially lower pressures, but poor LV function may increase afterload, leading to decreased output and pulmonary edema [150].

A study by Coromilas et al. compared percutaneous RVADs (pRVADs) and surgical RVADs (sRVADs) in 40 patients with acute right heart failure after LVAD implantation. While sRVADs showed higher flows (5.2 ± 0.9 L/min vs. 4.0 ± 0.4 L/min), improvements in hemodynamics were similar. The pRVAD group experienced shorter ventilation times, required fewer blood transfusions, and had reduced lengths of stay in intensive care units and hospitals [151].

#### 4.5.3. Impella RP

The Impella RP is a catheter-mounted axial flow device designed for percutaneous RV support. The 22F impeller is mounted on an 11F catheter that displaces blood from the RA into the PA, bypassing the right ventricle RV and offering immediate hemodynamic improvement in patients with refractory RV failure [150]. In the RECOVER RIGHT trial, which evaluated its utility in 12 patients with acute myocardial infarction and in 18 patients post-cardiac surgery, the CVP and cardiac index improved shortly after activation, leading to the successful weaning of inotropes and vasopressors [152]. Moreover, case reports indicate successful Impella RP use in managing pulmonary embolism-induced RV failure in conditions where heart–lung interaction is distinct (right ventricular dilation, interventricular septum shift, left ventricular diastolic dysfunction, and small left ventricle) [153,154]. Though beneficial in many scenarios, especially in patients with isolated RV failure, its long-term efficacy remains debated, particularly in cases of persistent RV failure due to mechanical ventilation and ARDS [155].

#### 4.5.4. TandemHeart RVAD

The TandemHeart RVAD (TH-RVAD) utilizes an extracorporeal centrifugal pump that moves blood from the RA to the PA through venous cannulas, directly bypassing the RV. Studies, such as the THRIVE study, have demonstrated the device’s efficacy in acute hemodynamic improvement across various etiologies of RHF [156]. This device has been especially useful in post-left ventricular assist device (LVAD) implantation and acute myocardial infarction-related RV failure. Although beneficial, in-hospital mortality remained high at 57%, particularly in older patients with biventricular failure [150]. A notable advantage of the TH-RVAD is its ability to incorporate an oxygenator, which can further assist patients with concurrent respiratory failure. However, it remains best suited for patients without significant pulmonary hypertension, where PA pressure could limit the flow and overall efficacy of the device.

#### 4.5.5. Protek Duo and Veno-Pulmonary ECMO (V-P ECMO)

The Protek Duo is a more recently introduced dual-lumen percutaneous cannula system designed for veno-pulmonary arterial (V-P) ECMO support. The 29F or 31F cannula is inserted through the right internal jugular vein, with its proximal end in the RA and distal end in the main PA, effectively bypassing the RV. This innovative approach enables the Protek Duo to act as both a bypass for RV support and an oxygenator if needed, mitigating RV dysfunction and preventing the need for more invasive surgical interventions [157]. The use of the Protek Duo has been documented across a range of RHF etiologies, including ARDS, COVID-19, and post-LVAD implantation.

Notably, in a study of six patients whose ECMO was reconfigured from veno-venous (V-V) ECMO to V-P ECMO using Protek Duo, significant reductions in vasopressor requirements, lactatemia, and norepinephrine dose were observed. Four out of six patients showed echocardiographic resolution of severe ACP and improved RV systolic function, highlighting its effectiveness in providing RV assistance [158]. Furthermore, in patients with ARDS due to COVID-19, Protek Duo showed a survival benefit of 59–89% across five studies compared to those treated with invasive ventilation or other ECMO configurations. Additionally, this device demonstrated a reduction in acute kidney injury (AKI) and the need for continuous renal replacement therapy (CRRT), marking it as a promising tool in critical care settings [157].

Protek Duo is particularly advantageous in terms of ease of placement, utilizing transesophageal echocardiogram (TEE) views for guidance and removal at bedside with only local analgesia. However, its long-term safety and efficacy remain under investigation, with reports of complications like right-sided infective endocarditis [158]. Despite these challenges, the survival rate for patients bridged to lung transplantation using Protek Duo was high, with one-year and two-year survival rates reaching 80% and 75%, respectively [159]. Case studies also illustrate its use in noncardiac conditions, such as refractory hypoxemia and drowning-related RV failure, demonstrating its versatility [160].

Notably, in cases of severe COVID-19 ARDS, data comparing veno-venous ECMO (V-V ECMO) and the TandemLife Protek Duo demonstrate that in-hospital mortality (52.4% vs. 11.1%, *p* = 0.008) and 30-day mortality (42.9% vs. 5.6%, *p* = 0.011) were significantly lower in patients treated with RVAD/ECMO. RVAD support at the time of ECMO initiation resulted in no secondary end-organ damage and higher in-hospital and 30-day survival rates compared to IMV. Therefore, right ventricular support should be prioritized in the management of severe COVID-19 ARDS to improve outcomes [161].

**Figure 4:**
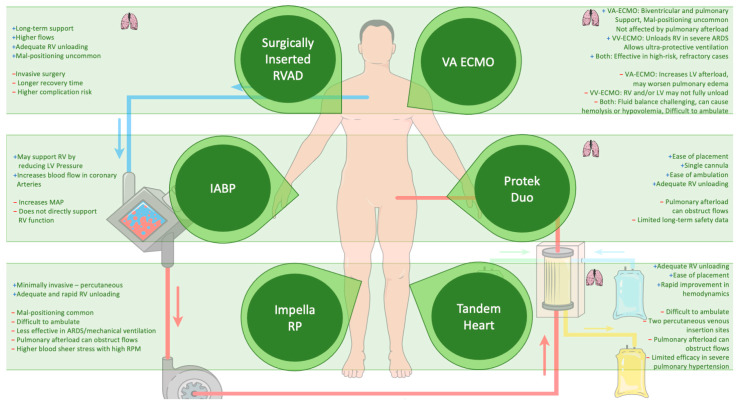
A comparative overview of mechanical circulatory support options: pros and cons. This figure provides a concise comparison of various mechanical circulatory support (MCS) options, including VA-ECMO, VV-ECMO, Impella RP, TandemHeart RVAD, Protek Duo, and Surgical RVAD. Each MCS modality is evaluated based on its effectiveness, invasiveness, and clinical applications. The advantages and limitations of each method are highlighted to assist in clinical decision making, emphasizing their utility in managing patients with critical cardiopulmonary failure and refractory hemodynamic instability. A small lung icon is placed near each MCS that supports the addition of an oxygenator, indicating its ability to provide respiratory support alongside hemodynamic stabilization. This visual helps clarify which systems can support both heart and lung functions when needed. (The + and − indicate the advantages and disadvantages of each modality). Abbreviations: ECMO (Extracorporeal Membrane Oxygenation), VA-ECMO (Veno-Arterial Extracorporeal Membrane Oxygenation), VV-ECMO (Veno-Venous Extracorporeal Membrane Oxygenation), MCS (Mechanical Circulatory Support), RVAD (Right Ventricular Assist Device), LVAD (Left Ventricular Assist Device), RV (Right Ventricle), LV (Left Ventricle), ARDS (Acute Respiratory Distress Syndrome), MAP (Mean Arterial Pressure). (Parts of the figure were drawn using pictures from Servier Medical Art. Servier Medical Art by Servier is licensed under a Creative Commons Attribution 4.0 Unported License (https://creativecommons.org/licenses/by/4.0/, accessed on 20 October 2024).

In summary, ECMO and other mechanical circulatory support (MCS) options, including the Impella RP, TandemHeart RVAD, and Protek Duo, provide essential life-saving support in critically ill patients with cardiopulmonary failure. They relieve the right ventricle by improving heart–lung interaction and balance the function of the right and left ventricles as well as the circulatory system. The choice of therapy—whether VV-ECMO, VA-ECMO, or specific devices—must be carefully tailored to each patient’s underlying pathophysiology, focusing on optimizing both right and left ventricular function while minimizing complications. Each device has distinct advantages: the Impella RP is effective for rapid RV unloading, TandemHeart serves patients with less severe pulmonary hypertension, and Protek Duo offers combined oxygenation and RV support. Ongoing research aims to refine these interventions, suggesting that percutaneous devices like the Protek Duo may significantly improve outcomes in RHF management, particularly for critically ill patients with concurrent respiratory failure, such as ARDS (Figure 4) (Table 4).

### 4.6. Machine Learning

Machine learning holds promise for predicting RV failure in patients undergoing MV and possibly will improve our understanding and management of heart–lung interactions. Recent studies have effectively utilized machine learning to forecast extubation success, demonstrating a sensitivity value in the range of 0.64 to 0.96 and specificity in the range of 0.73 to 0.85, with areas under the receiver operating characteristic curves ranging from 0.70 to 0.98 [162]. Key predictive features included the duration of MV, PaO_2_, blood urea nitrogen, heart rate, and Glasgow Coma Scale score. Additionally, machine learning models achieved an AUC of 0.71 for predicting cardiovascular hospitalizations or death, highlighting pulmonary artery pressure and the right ventricular end-diastolic volume as important predictors [163]. While further research is needed, the potential for improving outcomes in RV failure management is promising.

## 5. Key Points

- Positive-pressure mechanical ventilation alters pleural and transpulmonary pressures, affecting ventricular preload and afterload, which can lead to mainly right but also left ventricular dysfunction, especially in critical conditions.
- The interconnected dynamics of the right and left ventricles necessitate the careful management of ventilation settings to prevent hemodynamic instability in critically ill patients.
- Echocardiography is crucial for diagnosing and monitoring right ventricular function in critically ill patients, providing insights into RV dysfunction and its coupling with the pulmonary artery to inform clinical management.
- A hemodynamic assessment with arterial, central venous, and pulmonary artery catheters is essential for evaluating fluid responsiveness and right ventricular function in RHF, while effective RV failure management requires a multifaceted approach that includes fluid management, vasopressors, inotropes, and rhythm control.
- Optimizing mechanical ventilation for ARDS involves tailoring the PEEP and tidal volume to enhance both lung protection and right ventricular function. Our findings suggest that lower PEEP levels than recommended can improve hemodynamics, underscoring the need for individualized ventilation strategies.
- ECMO, particularly VA-ECMO and VV-ECMO, is essential for supporting patients with RHF and severe respiratory issues like ARDS, effectively improving right ventricular function and managing circulatory collapse.
- Managing RHF requires personalized approaches using devices like Impella RP for rapid unloading, TandemHeart for manageable pulmonary hypertension, and Protek Duo for combined oxygenation and RV support, with ongoing research to refine these interventions.

## 6. Conclusions

In conclusion, the intricate relationship between mechanical ventilation, right and left ventricular function, and hemodynamic stability in critically ill patients is paramount in the management of acute respiratory distress syndrome (ARDS) and right heart failure (RHF). Positive-pressure mechanical ventilation can adversely affect ventricular preload and afterload, potentially leading to ventricular dysfunction and cardiovascular collapse. Therefore, the careful monitoring and management of ventilation settings are crucial to prevent hemodynamic instability. Echocardiography and hemodynamic assessments play vital roles in diagnosing and monitoring right ventricular function, guiding clinical interventions. A multifaceted approach, including fluid management, vasopressors, and rhythm control, is essential for optimizing right ventricular performance. Furthermore, ECMO and various mechanical circulatory support devices, such as Impella RP, TandemHeart, and Protek Duo, provide critical support for patients facing RHF and severe respiratory complications. Ongoing research will enhance our understanding and application of these technologies, ultimately improving outcomes for this vulnerable patient population.

## Figures and Tables

**Table 1:** Intrathoracic pressures during unassisted breathing and mechanical ventilation (The plus (+) and minus (−) signs in the table represent whether the pressures are positive, negative, or variable under the two types of ventilation).

Pressure Type	Normal Ventilation (Negative Pressure Ventilation	Mechanical Ventilation (Possitive Pressure Ventilation)
Pleural Pressure (Ppl)	−	+/−
Alveolar Pressure (Palv)	−	+
Airway Pressure (Paw)	−	+
Transpulmonary Pressure (TTP)	+/−	+

**Table 2:** A comparative evaluation of echocardiographic parameters for assessing right ventricular function in critical illness. This table evaluates various echocardiographic parameters used to assess right ventricular (RV) function in critically ill patients. The parameters include RV end-diastolic area/left ventricular end-diastolic area ratio. The plus (+) and minus (−) symbols in the table indicate whether a specific echocardiographic parameter meets a criterion (+) or does not meet it (−). (RVEDA/LVEDA), RV fractional area change (RVFAC), tricuspid annular plane systolic excursion (TAPSE), systolic velocity of the tricuspid annulus (RV S′), RV longitudinal strain (RV-LS), RV outflow tract velocity–time integral (RVOT VTI), 3D echocardiography, tricuspid regurgitation (TR) assessment, inferior vena cava (IVC) size, RV–pulmonary artery (PA) coupling (FACRV/RVSP, TAPSE/RVSP), RV load–-adaptation index (LAIRV), and the Venous Excess Ultrasound Score (VEXUS). Each parameter’s advantages and limitations in clinical application are summarized.

ECHO Parameter	Easy to Calculate	Reflects RV Function Well	Sensitive for Early Detection	Independent of Geometric Assumption	Operator Dependent	Requires Good Image Quality	Non-Invasive	Useful in Emergency Settings	Applicability in Critical Illness	Load Dependence
RVEDA/LVEDA Ratio	+	−	−	−	−	+	+	+	+	−
RV Fractional Area Change (RVFAC)	+	+	−	+	−	+	+	+	+	+
TAPSE	+	+	−	−	+	+	+	+	+	+
RV S′	−	+	−	−	+	+	+	+	+	+
RV Longitudinal Strain (RV-LS)	−	+	+	−	+	−	+	−	+	−
RVOT Velocity–Time Integral (RVOT_VTI_)	−	+	+	−	−	+	+	+	+	−
3D Echocardiography of RVEF	−	+	+	+	+	−	−	−	+	−
Tricuspid Regurgitation (TR) Assessment	+	+	+	−	−	+	+	+	+	+
Inferior Vena Cava (IVC) Size and Flow	+	−	−	+	−	+	+	+	+	+
RV-PA Coupling (FACRV/RVSP, TAPSE/RVSP)	−	+	+	−	−	−	+	−	+	+
RV Load–Adaptation Index (LAIRV)	+	+	+	+	−	+	+	+	+	+
Venous Excess Ultrasound Score (VEXUS)	+	+	-	-	-	+	+	+	+	-

**Table 3:** Key indicators, diagnostic approaches, and hemodynamic monitoring in acute right heart failure in mechanically ventilated patients.

Aspect	Key Indicators/Diagnostic Approaches	Details
Clinical Presentation	Symptoms of Acute RHF	Increased oxygen demands, cardiovascular collapse, arrhythmias, elevated jugular venous pressure, gallop rhythm, systolic murmur, organomegaly, deep venous thrombosis (especially in venous thromboembolism-related RHF), persistent weaning failure from mechanical ventilation, mismatch between right ventricular dysfunction and ventilatory support, especially with left ventricular dysfunction
Chest X-ray (CXR)	Radiological Findings	Enlarged main pulmonary artery, regional oligemia (in massive pulmonary embolism); CXR mainly used to exclude conditions mimicking RHF (e.g., pleural effusions, atelectasis, pulmonary edema, pneumothorax)
Electrocardiogram (ECG)	ECG Indicators of RHF	Qr pattern in lead V1, right bundle branch block (R duration > 100 ms in V1), T wave inversions (V1–V4), S1Q3T3 pattern, acute Q waves in V1–V3 or right-sided Q waves in V3R–V6R (suggesting right ventricular infarction) Though specific, ECG has limited sensitivity for diagnosing RHF
Hemodynamic Monitoring (Pulse Pressure Variation, PPV)	Monitoring Fluid Responsiveness	PPV helps assess preload dependence and fluid responsiveness, especially in ARDS; high PPV (>12–13%) suggests fluid responsiveness; tidal volume challenge and PEEP changes can enhance predictive accuracy; PPV is less reliable with low lung compliance or spontaneous breathing
Central Venous Catheter (CVC)	CVP Monitoring for RV Function	CVP trends provide insights into RV function and fluid responsiveness; rapid rise in CVP during volume loading without stroke volume improvement suggests right heart dysfunction; limited accuracy in conditions like pulmonary hypertension and when pleural pressure is elevated
Echocardiography (ECHO)	Evaluation of RV and LV Function	Non-invasive assessment of RV size, function, and pulmonary vascular resistance; key techniques include measuring RV end-diastolic area (RVEDA), right ventricular fractional area change (RVFAC), tricuspid annular plane systolic excursion (TAPSE), RV longitudinal strain (RV-LS), and RV-PA coupling (TAPSE/PASP ratio)
Advanced Echocardiography	Advanced Imaging Techniques	3D echocardiography provides volumetric assessment for LV systolic function; RV-LS via 2D speckle tracking echocardiography (STE) is sensitive for early detection of RV dysfunction, even with normal RV ejection fraction TAPSE/PASP ratio correlates with outcomes
Fluid Responsiveness and Volume Status	Inferior and Superior Vena Cava (IVC/SVC)	SVC collapsibility > 36% and IVC collapsibility > 12% are useful for predicting fluid responsiveness in mechanically ventilated patients
Pulmonary Artery Catheter (PAC)	Advanced Hemodynamic Monitoring	PAC helps estimate true LV filling pressures, pulmonary artery pressures (PAP), pulmonary vascular resistances, and mixed venous oxygen saturation (SvO_2_); valuable for monitoring RV dysfunction, particularly in severe ARDS, and evaluating responses to therapy
Monitoring in ARDS and RV Dysfunction	Monitoring RV Function in ARDS	Use of PAC and CVC, alongside PPV, is essential for assessing fluid responsiveness and RV function in patients with ARDS; elevated CVP relative to PAOP may indicate RV failure

Abbreviation list: CVP—Central Venous Pressure; PAP—Pulmonary Artery Pressure; PAOP—Pulmonary Artery Occlusion Pressure.

**Table 4:** Summary of management options for right heart failure in mechanically ventilated patients.

Aspect	Details
Fluid Therapy	- Preload: RV relies on preload; fluid loading can worsen pulmonary edema and cor pulmonale - Fluid Responsiveness: Difficult to assess in ARDS due to impaired heart–lung interactions; cautious resuscitation is recommended for hypovolemic patients - CVP: Debate on its reliability to guide therapy; better assessed with transmural CVP
Pharmaceutical Treatment	
Vasopressors	- Norepinephrine: Improves RV function by elevating MAP and RV supply; risks at high doses: increased RV afterload and PVR - Epinephrine: Alternative for severely compromised RV function
Inotropes	- Dobutamine: Enhances RV contractility in ARDS; high doses may increase PVR and tachycardia - PDE III Inhibitors (Milrinone): Improves RV function, especially in high pulmonary afterload - Levosimendan: Calcium sensitizer; improves hemodynamics without increasing oxygen demand
Vasodilators	- Inhaled Nitric Oxide: Pulmonary vasodilation with minimal systemic effects; useful in ARDS and pulmonary embolism - Sildenafil: PDE-5 inhibitor; may improve symptoms in chronic PAH but requires more evidence for acute settings
Diuretics	Use in RV overload, but may cause cardio-renal syndrome; CVVH may be more beneficial than diuretics in patients resistant to them
Ventilation Strategies	
Rhythm Control	- Synchronizing right atrium and RV is key - Temporary pacing may improve cardiac output in certain cases
PEEP and Lung Recruitment	- PEEP prevents alveolar collapse, improves oxygenation, and plays crucial role in stabilizing ARDS patients - Excessive PEEP can worsen RV function
Pulmonary Vasoconstriction	- Hypoxia and hypercapnia lead to pulmonary vasoconstriction, increasing RV afterload - Mechanical ventilation should aim for normoxia and normocapnia with lungand heart-protective strategies
PEEP Optimization and RV Afterload	- Optimizing PEEP helps enhance alveolar patency and reduces RV afterload - High PEEP levels (>15 cmH_2_O) can worsen RV systolic function, while lower PEEP levels have been shown to reduce RV dilation and improve RV function in patients with ARDS, particularly those with COVID-19-induced ARDS (CARDS).
Individualized PEEP Settings	PEEP settings should be personalized based on factors like lung compliance, functional residual capacity, PaCO_2_, RV function, and systolic pulmonary artery pressure (via tricuspid regurgitation) - Overzealous adherence to high PEEP recommendations may not always align with patient-specific needs, especially in patients with COVID-19-induced ARDS exhibiting higher RV dysfunction
Prone Positioning and RV Function	- Prone positioning improves ventilation uniformity and may unload the RV - Studies have shown that PP restores RV function in overloaded patients - Improved oxygenation after PP is often attributed to reduced shunt fraction rather than hemodynamic changes
PEEP and Left Ventricular Function	- By increasing intrathoracic pressure, PEEP reduces LV transmural pressure, which can lower afterload and improve cardiac output - This effect is beneficial in conditions like cardiogenic pulmonary edema and is supported by use of CPAP - When carefully titrated, PEEP can optimize both respiratory and cardiovascular function
Mechanical Circulatory Support (MCS) (Figure 3)	- MCS may be required in critically ill patients when conventional therapies fail - VA-ECMO, often combined with devices like LV-Impella or RV-Impella, is recommended by ESC for patients with refractory circulatory collapse or cardiac arrest - VA-ECMO is also recommended for selected patients with pulmonary hypertension as bridge to transplantation or recovery - Use of IABP in cardiogenic shock is debated, especially in RV or biventricular failure cases, where more advanced MCS might be needed - Decision to use RVAD depends on managing RV afterload or filling limitations, with caveat that elevated LVEDP can exacerbate pulmonary edema, making LVAD better - Careful volume management is essential

Abbreviation list: ARDS—Acute Respiratory Distress Syndrome; CVP—Central Venous Pressure; MAP—Mean Arterial Pressure; PVR—Pulmonary Vascular Resistance; PAH—Pulmonary Arterial Hypertension; PDE—Phosphodiesterase; PDE III—Phosphodiesterase Type III; RV—Right Ventricle; RVAD—Right Ventricular Assist Device; LVAD—Left Ventricular Assist Device; IABP—Intra-Aortic Balloon Pump; VA-ECMO—Veno-Arterial Extracorporeal Membrane Oxygenation; LV-Impella—Left Ventricular Impella; RV-Impella—Right Ventricular Impella; CPAP—Continuous Positive Airway Pressure; MCS—Mechanical Circulatory Support; CVVH—Continuous Veno-Venous Hemofiltration; PaCO_2_—Partial Pressure of Carbon Dioxide in Arterial Blood; MAP—Mean Arterial Pressure.
